# Causal relevance of circulating high-density lipoprotein cholesterol with cancer: a Mendelian randomization meta-analysis

**DOI:** 10.1038/srep09495

**Published:** 2015-03-30

**Authors:** Chunhua Yang, Geng Tian, Jia Mi, Xiaodan Wei, Xuri Li, Xianglin Li, Wenming Wang, Bin Wang

**Affiliations:** 1 Medicine and Pharmacy Research Center, Binzhou Medical University, Laishan District, Yantai, Shandong, China

## Abstract

We summarized published data on the associations of apolipoprotein E (*APOE*) gene ε2/ε3/ε4 polymorphism with both cancer risk and circulating lipid profiles, aiming to examine the causal relevance between lipids and cancer risk. Article identification and data abstraction were conducted in duplicate and independently by two authors. Data were analyzed by STATA software. Twenty-five articles that examined the associations of *APOE* gene ε2/ε3/ε4 polymorphism with either cancer risk (n = 22) or circulating lipid changes (n = 4) were eligible. The presence of ε2 and ε4 alleles showed no overall associations with overall cancer risk when compared with ε3 allele. The ε4 allele was significantly associated with 1.40-fold (odds ratio or OR = 1.40; 95% confidence interval or CI: 1.00–1.94; P = 0.047) increased risk of developing cancer in Asian populations, and the presence of heterogeneity was low (*I*^2^ = 37.6%). Carriers of ε3/ε4 genotype had a significant reduction in circulating HDL-C (WMD = −2.62; 95% CI: −4.19 to −1.04; P = 0.001) without heterogeneity (*I*^2^ = 16.6%). The predicted odds of having cancer for 1 mg/dL reduction in circulating HDL-C was 1.14 (95% CI: 1.00 to 1.89). The findings of this Mendelian randomization meta-analysis demonstrate that reduced circulating HDL-C might be a potentially causal risk factor for the development of overall cancer in Asians.

Some observational studies have revealed that people with low circulating cholesterol level tended to be more susceptible to many malignancies, such as lung cancer and breast cancer^1^,^2^. As a central regulator in cholesterol metabolism, apolipoprotein E (APOE) is increasingly recognized as playing a potent inhibitory role in angiogenesis and cancer cell growth^3^. It has been estimated that close to 60% of circulating cholesterol variation is under genetic control, and thereof 14% variation is attributable to *APOE* genetic defects^4^. The genomic sequence of human *APOE* (gene ID: 348, 19q13.2) is polymorphic at two nucleotides, which yields 3 alleles (ε2, ε3, ε4) and 6 genotypes (ε2/ε2, ε2/ε3, ε3/ε3, ε2/ε4, ε3/ε4, ε4/ε4), with diverse receptor-binding capabilities^5^. As evidenced, this capability was proven to be defective for the ε2 allele with its carriers exhibiting lower circulating cholesterol level and higher triglyceride level when compared with ε3 homozygotes; in contrast, circulating total cholesterol and low-density lipoprotein cholesterol appear to be higher in those with ε4 allele^6^. In spite of exhaustive investigations, published data on the associations between *APOE* gene ε2/ε3/ε4 polymorphism and cancer risk are conflicting and inconclusive^5^,^7^,^8^,^9^. A recent meta-analysis by Anand et al who examined this association in 16 studies failed to detect any positive signal except in cohort studies^9^. However, they did not compare the changes of circulating lipid levels across *APOE* gene ε2/ε3/ε4 genotypes, which would be of importance to provide background data to infer causality between circulating lipids and cancer risk. To fill this gap in knowledge and generate added information, we revisited this topic and summarized the associations of *APOE* gene ε2/ε3/ε4 polymorphism with both cancer risk and circulating lipid profiles in a large meta-analysis implementing Mendelian randomization technique.

## Results

### Eligible articles

Of 530 potentially relevant articles identified according to our search strategy, 25 articles that examined the associations of *APOE* gene ε2/ε3/ε4 polymorphism with either cancer risk (n = 22) or circulating lipid changes (n = 4) were eligible according to the predefined inclusion and exclusion criteria^5^,^7^,^8^,^10^,^11^,^12^,^13^,^14^,^15^,^16^,^17^,^18^,^19^,^20^,^21^,^22^,^23^,^24^,^25^,^26^,^27^,^28^,^29^,^30^,^31^. The first article was published in 1996^10^. The total sample size ranged from 78 in McDonald et al study^31^ to 74033 in Benn et al study^27^. For 22 *APOE*-cancer association articles with 26 independent studies, there were 13478 cancer patients and 77592 controls in total. For 4 *APOE*-lipids association studies, data provided in both cancer patients and controls were analyzed separately, resulting in 6 independent studies for TG and 7 studies respectively for TC, HDL-C and LDL-C.

### Host characteristics

Baseline host characteristics of study populations for the associations of *APOE* gene ε2/ε3/ε4 polymorphism with cancer risk and circulating lipid changes are presented in Table 1 and Table 2, respectively.

For 26 *APOE*-cancer association studies, breast cancer was reported in 10 studies, colorectal cancer in 8 studies, multiple cancers in 3 studies, prostate cancer in 2 studies, gastric, head and neck, hepatocellular cancers respectively in 1 study. 14 of 26 studies were conducted in White populations, and 4 respectively in Asian, Latinos and mixed populations. As for source of controls, 10 studies enrolled population-based controls, and 16 studies enrolled hospital-based studies. The majority of 26 studies were retrospective in design (n = 23) with the rest being prospective (n = 3). Cancer patients and controls were reported to be matched in 10 studies, unmatched in 10 studies and unreported in 6 studies. The mean age was significantly higher in cancer patients than in controls (58.07 years versus 52.69 years, P = 0.001). No significance was observed in gender, BMI, smoking and family history of cancer between the two groups (P > 0.05).

### Overall comparisons for cancer risk

**Figure 1:**
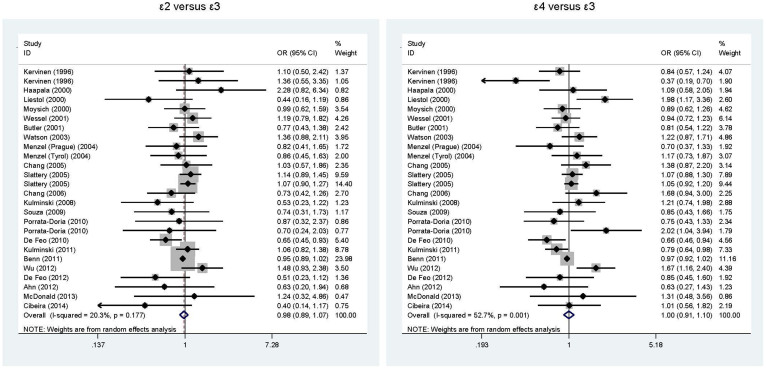
Overall comparisons of *APOE* gene ε2 versus ε3 (the left) and ε4 versus ε3 (the right) in association with cancer risk.

**Figure 2:**
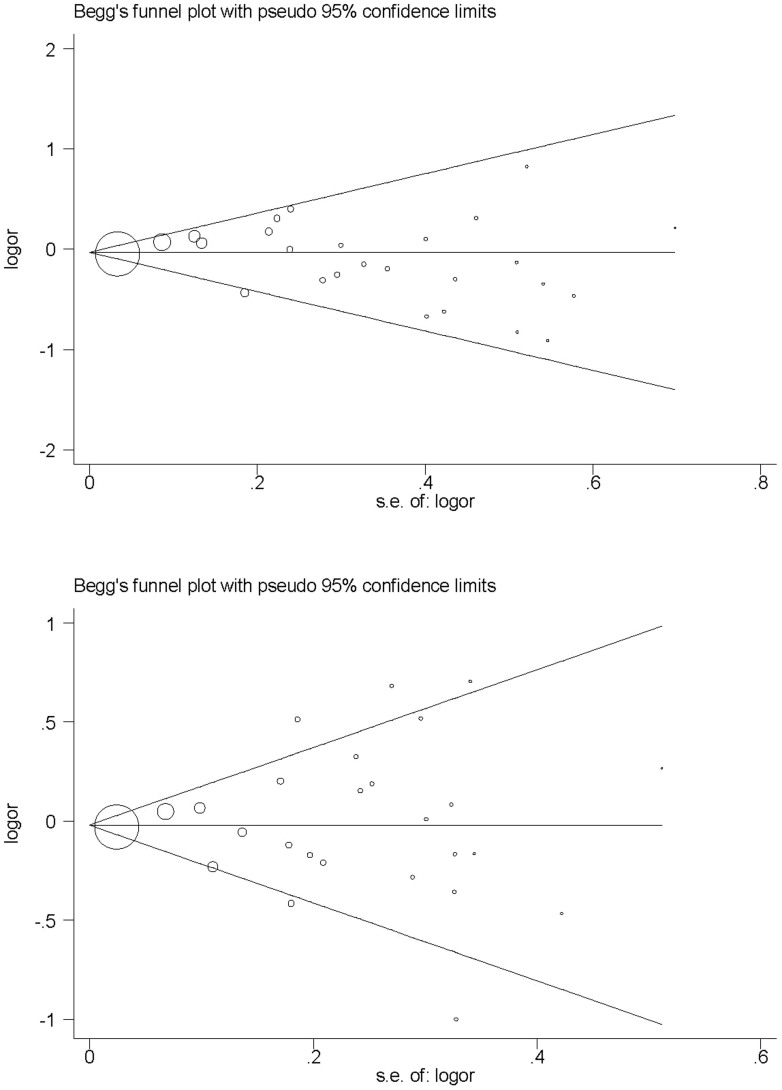
Begg's funnel plots for the comparisons of *APOE* gene ε2 versus ε3 (the upper) and ε4 versus ε3 (the lower).

Considering the low numbers of *APOE* gene ε2/ε2, ε2/ε4, ε4/ε4 genotypes, only allelic comparisons (ε2 versus ε3 and ε4 versus ε3) were computed. As shown in Figure 1, the presence of ε2 and ε4 alleles showed no overall associations with overall cancer risk when compared with ε3 allele. There was no evidence of heterogeneity for the comparison of ε2 with ε3 (*I*^2^ = 20.3%), but significant heterogeneity for the comparison of ε4 with ε3 (*I*^2^ = 20.3%). The low probabilities of publication bias for both comparisons were reflected by the Begg's funnel plots (Figure 2) and Egger's tests (P = 0.512 for ε2 with ε3 and 0.662 for ε4 with ε3). The trim and fill method indicated that only one missing study was required for the comparison of ε4 with ε3 to make the Filled funnel plot symmetrical (Supplementary Figure S1).

### Sensitivity analysis

The direction and magnitude of pooled effect estimates regarding the comparisons of *APOE* gene ε2 and ε4 alleles with ε3 allele were confirmed in our sensitivity analysis, respectively.

### Stratified comparisons for cancer risk

In an attempt to examine whether risk prediction was heterogeneous between different subgroups, several subgroup analyses were conducted according to cancer type, ethnicity, source of controls, study design, matched status and sample size, respectively (Table 3). There was no indicative of significant associations for the comparisons of ε2 versus ε3 and ε4 versus ε3 across all subgroups except for the latter comparison in Asians. The ε4 allele was significantly associated with 1.40-fold (OR = 1.40; 95% CI: 1.00–1.94; P = 0.047) increased risk of developing cancer in Asian populations, and the presence of heterogeneity was low (*I*^2^ = 37.6%), as compared with 8% reduced risk in Caucasian populations (OR = 0.92; 95% CI: 0.81–1.03; P = 0.135).

### Meta-regression analysis

As age, gender, BMI, smoking and family history of cancer were continuous, several meta-regression models were constructed by including them as covariates separately, and still no significance was attained.

### Overall comparisons for lipid changes

**Figure 3:**
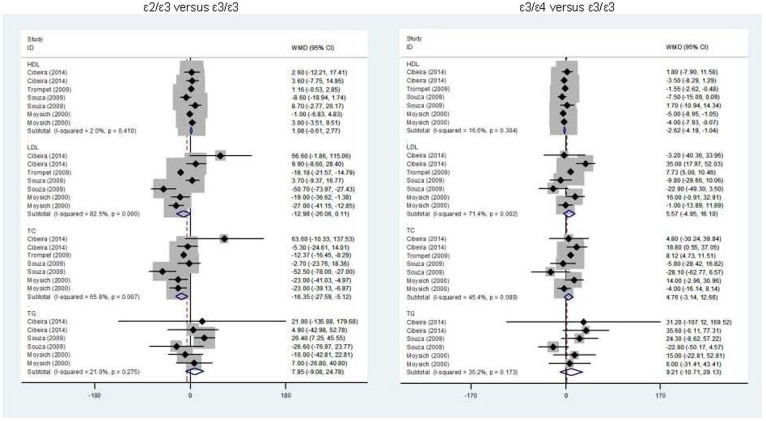
Overall lipid changes for the comparisons of *APOE* gene ε2/ε3 versus ε3/ε3 (the left) and ε3/ε4 versus ε3/ε3 (the right).

In view of limited data on *APOE* gene ε2/ε2, ε2/ε4, ε4/ε4 genotypes, mean lipid changes were only compared for genotype ε2/ε3 versus ε3/ε3 and ε3/ε4 and ε3/ε3 (Figure 3). Out of four lipids (TG, TC, HDL-C and LDL-C) examined, carriers of ε2/ε3 genotype had a significant reduction in circulating TC (WMD = −16.35; 95% CI: −27.59 to −5.12; P = 0.004) when compared with those with ε3/ε3 genotype, yet with strong evidence of heterogeneity (*I*^2^ = 65.8%). In contrast, carriers of ε3/ε4 genotype had a significant reduction in circulating HDL-C (WMD = −2.62; 95% CI: −4.19 to −1.04; P = 0.001) without heterogeneity (*I*^2^ = 16.6%). No statistical significance was observed for the other comparisons.

### Causal prediction of circulating lipids for cancer

At the requirements of Mendelian randomization technique, causal relevance between circulating lipids and cancer risk was only calculated based on the association between *APOE* gene ε4 allele and cancer risk in Asians and the relationship between ε3/ε4 genotype and circulating HDL-C reduction. The predicted odds of overall cancer for 1 mg/dL reduction in circulating HDL-C was 1.14 (95% CI: 1.00 to 1.89), and this estimate was significant at a significance level of 5% as the null hypothesis value of 1 was not included by the estimated 95% CI.

## Discussion

Extending the findings of a recent meta-analysis by Anand et al,^9^ we through a larger Mendelian randomization meta-analysis of the data from 25 articles and on 91070 participants, found that reduced circulating HDL-C might be a potentially causal risk factor for the development of overall cancer in Asians by using *APOE* gene ε2/ε3/ε4 polymorphism as a surrogate marker. This meta-analysis is unique to our knowledge, as it is to date the first to address the causal relevance between circulating lipids and cancer risk in medical literature.

Several observational and clinical studies have demonstrated an inverse association between circulating HDL-C and cancer risk; however, this association is currently subject to an ongoing debate, as the issues of confounding and reverse causation are intractable in classic epidemiology. Fortunately, Mendelian randomization has been introduced as a viable technique to overcome drawbacks of observational studies and obtain robust causal estimates^32^. Recently, a large-scale prospective study that examined the association of HDL-C with cancer incidence in patients with type II diabetes demonstrated that this significant association might be attributable to confounding and reverse causation^33^. Another prospective study by Kucharska-Newton et al identified a relatively weak inverse association between HDL-C and lung cancer, and this association was dependent on smoking status^1^. It is widely believed that circulating HDL-C is under considerable genetic control with heritability estimates of up to 60%^4^,^34^. Several lines of evidence supported a close relation between *APOE* genetic alterations and circulating HDL profiles^7^,^35^,^36^,^37^, which was mirrored in the current meta-analysis revealing that the presence of *APOE* gene ε4 allele was associated with significantly reduced HDL-C in circulation, reinforcing the soundness of selecting ε2/ε3/ε4 polymorphism as a surrogate marker. Besides, we observed that the ε4 allele was particularly overrepresented in Asian cancer patients relative to controls. Based on these observations, it is reasonably expected that low circulating HDL-C conferred by *APOE* gene ε4 allele is causally related with an increased risk of cancer in Asians. Nevertheless, given the inadequate statistical power of this meta-analysis in subgroup analyses, far larger sample sizes than examined here will be required to produce sufficient power to evaluate the causality between circulating HDL-C and cancer risk.

Several limitations of the present meta-analysis need to be acknowledged. Firstly, we restricted our search scope to published articles written in only English language, and we cannot totally rule out the likelihood of selective publication bias. Secondly, almost all involved studies had circulating lipids measured only once, which cannot reflect its long-term profile in the development of cancer. Thirdly, this meta-analysis was based on summarized data, rather than individual participant data, precluding further gene-to-environment interactions. Fourthly, only *APOE* gene ε2/ε3/ε4 polymorphism was selected in this study, and investigations on other candidate genes or polymorphisms involved in HDL-C regulation were highly encouraged, leaving a challengeable task to test whether this polymorphism integrated with other risk determinants will enhance cancer risk prediction. Fifthly, one key assumption of Mendelian randomization is that the genetic polymorphism under study should not exhibit a pleiotropic effect, which is beyond our capability in this meta-analysis to eliminate this effect. Nevertheless, the present meta-analysis enriched our understandings of circulating HDL-C in molecular carcinogenesis, which would facilitate the identification of at-risk individuals who would develop cancer later in future clinical screening.

Taken together, the findings of this Mendelian randomization meta-analysis demonstrate that reduced circulating HDL-C might be a potentially causal risk factor for the development of overall cancer in Asians. For practical reasons, it is encouraging to deem this study as a beginning instead of an endpoint of investigations to establish and optimize the background data to understanding the causal relevance of circulating HDL-C to carcinogenesis of multiple solid tumors.

## Methods

The present meta-analysis was carried out in accordance with the guidelines formulated in the PRISMA (Preferred Reporting Items for Systematic Reviews and Meta-analyses) statement (see the Supporting Checklist)^38^.

### Search strategy

To identify all relevant articles that assessed the associations of *APOE* gene ε2/ε3/ε4 polymorphism with cancer risk or circulating lipid changes, we systematically searched PubMed and Embase electronic databases as of December 20, 2014 using the following subject terms, ‘apolipoprotein E or apo E or APOE or apo-E′, in combination with ‘cancer or carcinoma or neoplasia or tumor or adenoma or neoplasm or myeloma or melanoma or lymphoma or leukaemia or leiomyoma’ and ‘polymorphism or variant or variation or mutation or genotype or allele or SNP’. We also manually checked the reference lists of major original articles and reviews for the missing citations of relevance.

The titles and abstracts of all retrieved articles were independently read by two authors of this meta-analysis (Chunhua Yang and Xuri Li) to assess their eligibility. If we cannot reject an article with certainty, its full text was reviewed to ascertain whether relevant data were provided and if necessary we contacted study authors by emails to request additional information. We extracted data from the most recent or complete article if a same study group was reported by more than one publications. This process was run in duplicate and independently by the same two authors, and any uncertainty over the eligibility was adjudicated by a discussion or further joint inspection of original articles.

### Inclusion/exclusion criteria

All studies that met the following criteria were included: (a) regarding cancer risk, data on associations between *APOE* gene ε2/ε3/ε4 polymorphism and all sites of cancer except for skin were provided; (b) regarding circulating lipid changes, the mean or medium values and the corresponding standard deviation of circulating lipids including triglyceride (TG), total cholesterol (TC), high-density lipoprotein cholesterol (HDL-C) and low-density lipoprotein cholesterol (LDL-C) were provided across *APOE* gene ε2/ε3/ε4 alleles or genotypes; (c) study design should be either prospective or retrospective; (d) detailed genotype or allele counts of *APOE* gene ε2/ε3/ε4 polymorphism were tractable between cancer patients and controls.

Conference abstracts or proceedings that did not specifically address the topic of our analysis were excluded from full-text review. Case reports or series, editorials, narrative or systematic reviews, or non-English articles were also not covered. Also this meta-analysis did not involve studies that examined the progression, severity or response to treatment or survival of cancer in association with *APOE* gene ε2/ε3/ε4 polymorphism or that were lack of cancer-free controls.

### Data gathering

Data were gathered independently from each eligible article by two authors (Chunhua Yang and Xuri Li) according to a predefined protocol developed by all contributing authors, including the first author's last name, publication year, ethnicity, cancer subtype, case-control matched status, source of controls, study design, sample size, the genotype and/or allele counts of *APOE* gene ε2/ε3/ε4 polymorphism between cancer patients and controls, the mean or medium (standard deviation) values of circulating TG, TC, HDL-C and LDL-C for each *APOE* gene ε2/ε3/ε4 allele or genotype carriers, as well as baseline characteristics of study populations when available such as age, gender, body mass index (BMI), smoking and the family history of cancer. The units of circulating TG, TC, HDL-C and LDL-C were uniformly standardized as mg/dL for consistency.

### Statistics

All statistical analyses were managed with the use of STATA software (StataCorp, Texas, USA, version 12.0) on Windows.

The association of *APOE* gene ε2 or ε4 allele with cancer risk was expressed as odds ratio (OR) and 95% confidence interval (95% CI) when compared with the ε3 allele. Considering the confounding effect of heterogeneity between studies, only random-effects model with the DerSimonian & Laird method^39^ was employed.

The probability of publication bias was assessed by visual Begg's funnel plot and the Egger's test, as well as the trim-and-fill method which can infer the existence of unpublished hidden articles from a filled funnel plot and correct the meta-analysis by imputing the presence of missing studies to yield an unbiased pooled estimate.

Heterogeneity was quantified by the inconsistency index (*I*^2^) statistic, which ranges from 0% to 100% and is defined as the percentage of the observed between-study variability that is due to heterogeneity rather than chance. In this meta-analysis, *I*^2^ > 50% is designated as a threshold to indicate significant heterogeneity^39^. To identify potential sources of heterogeneity, predetermined subgroup analyses and meta-regression analyses were performed to model categorical and continuous host characteristics, respectively. For meta-regression analysis, given that some host characteristics had a lot of missing values such as smoking, each characteristic was modeled separately.

To evaluate the impact of individual studies on pooled effect estimates, we performed sensitivity analysis by sequentially omitting each study one at a time and computing differential estimates for remaining studies.

Under the assumptions of Mendelian randomization as formulated by Katan MB in 1986^40^, we calculated the risk prediction as the ratio of the coefficient of the association between *APOE* gene ε2/ε3/ε4 polymorphism and cancer risk to that of the relationship between this polymorphism and circulating lipid changes to reflect the possible causal relevance of these lipids on cancer.

## Author Contributions

G.T., J.M. and B.W. conceived and designed the experiments; C.Y. and XuriL performed the experiments; C.Y. and G.T. analyzed the data; X.W., XuriL, XianglinL and W.W. contributed materials/analysis tools; C.Y., G.T. and B.W. wrote and revised the manuscript. All authors reviewed and approved the manuscript prior to submission.

## Supplementary Material

Supplementary Information Supplementary materials

## Figures and Tables

**Table 1:** Baseline characteristics of all study populations

Author (year)	Cancer type	Race	Source	Design	Match	Sample size		Age (years)		Males		BMI (kg/m^2^)		Smoking		Family history	
						Cases	Cont's	Cases	Cont's	Cases	Cont's	Cases	Cont's	Cases	Cont's	Cases	Cont's
Cibeira (2014)	Breast	Latinos	PB	PS	YES	47	165	57.6	56.1	0.00	0.00	28.2	28.9	NA	NA	NA	NA
McDonald (2013)	Breast	White	HB	RS	NA	54	24	51.2	47.0	0.00	0.00	NA	NA	NA	NA	NA	NA
Ahn (2012)	Hepatocellular	Asian	PB	RS	NO	59	47	53.9	45.3	0.76	0.66	NA	NA	NA	NA	NA	NA
De Feo (2012)	Gastric	White	HB	RS	NO	156	444	67.1	59.0	0.53	0.59	NA	NA	0.49	0.46	0.38	0.29
Wu (2012)	Breast	Asian	HB	RS	NO	306	300	48.5	41.3	0.00	0.00	NA	NA	NA	NA	NA	NA
Benn (2011)	All	White	PB	PS	YES	6816	67217	NA	NA	NA	NA	NA	NA	NA	NA	NA	NA
Kulminski (2011)	All	Mixed	PB	PS	NA	701	895	NA	NA	NA	NA	NA	NA	NA	NA	NA	NA
De Feo (2010)	Head and neck	White	HB	RS	NO	417	436	63.1	59.3	0.81	0.58	NA	NA	0.86	0.43	0.31	0.21
Porrata-Doria (2010)	Breast	Latinos	HB	RS	YES	63	106	43.0	41.7	0.00	0.00	26.6	26.5	0.14	0.12	0.43	0.49
Porrata-Doria (2010)	Breast	Latinos	HB	RS	YES	142	123	63.8	61.8	0.00	0.00	27.8	27.9	0.16	0.13	0.35	0.40
Souza (2009)	Colorectal	Latinos	HB	RS	YES	87	73	60.6	61.6	0.47	0.44	NA	NA	0.49	0.48	NA	NA
Kulminski (2008)	Colorectal	White	PB	RS	YES	77	1644	NA	NA	NA	NA	NA	NA	NA	NA	NA	NA
Chang (2006)	Breast	Asian	HB	RS	YES	291	148	49.5	50.9	0.00	0.00	NA	NA	NA	NA	NA	NA
Slattery (2005)	Colorectal	Mixed	PB	RS	YES	1556	1948	NA	NA	0.56	0.53	27.8	26.8	NA	NA	0.16	0.09
Slattery (2005)	Colorectal	Mixed	PB	RS	YES	777	988	NA	NA	0.59	0.57	27.8	27.4	NA	NA	0.11	0.08
Chang (2005)	Breast	Asian	HB	RS	NO	290	232	47.4	40.2	0.00	0.00	NA	NA	NA	NA	NA	NA
Menzel (2004)	Breast	White	PB	RS	NO	220	400	56.0	39.0	0.00	0.00	NA	NA	NA	NA	NA	NA
Menzel (2004)	Breast	White	PB	RS	NA	190	231	58.0	60.0	0.00	0.00	NA	NA	NA	NA	NA	NA
Watson (2003)	Colorectal	White	HB	RS	NO	206	353	68.7	60.6	0.60	0.67	NA	NA	0.41	0.50	NA	NA
Butler (2001)	Colorectal	White	HB	RS	NO	167	200	70.0	51.0	0.52	0.52	NA	NA	NA	NA	NA	NA
Wessel (2001)	Prostate	White	HB	RS	NA	230	798	NA	NA	NA	NA	NA	NA	NA	NA	NA	NA
Moysich (2000)	Breast	White	PB	RS	YES	260	332	56.9	58.0	0.00	0.00	25.5	25.5	NA	NA	0.16	0.08
Liestol (2000)	All	Mixed	HB	RS	NA	71	126	NA	NA	NA	NA	NA	NA	NA	NA	NA	NA
Haapala (2000)	Prostate	White	HB	RS	NA	38	163	NA	NA	1.00	1.00	NA	NA	NA	NA	NA	NA
Kervinen (1996)	Colorectal	White	HB	RS	NO	122	199	67.2	57.8	0.44	0.88	NA	NA	NA	NA	NA	NA
Kervinen (1996)	Colorectal	White	HB	RS	NO	135	199	62.9	57.8	0.62	0.88	NA	NA	NA	NA	NA	NA

Abbreviations: PB, population-based; HB, hospital-based; PS, prospective; RS, retrospective; BMI, body mass index; NA, not available.

**Table 2:** Distributions of circulating lipids across *APOE* gene ε2/ε3/ε4 genotypes in all qualified studies

Author (year)	Cancer type	Race	Status	Lipids (mg/dL)	ε2/3		ε3/3		ε3/4	
					Mean	SD	Mean	SD	Mean	SD
Cibeira (2014)	Breast	Latinos	Cases	HDL	51.00	14.40	48.40	12.10	50.20	15.20
Cibeira (2014)	Breast	Latinos	Controls	HDL	57.20	22.70	53.60	11.10	50.10	13.60
Trompet (2009)	All types	White	Both	HDL	50.66	14.09	49.50	16.26	47.95	9.74
Souza (2009)	Colorectal	Latinos	Cases	HDL	33.30	8.30	41.90	16.10	34.40	13.50
Souza (2009)	Colorectal	Latinos	Controls	HDL	52.10	15.40	43.40	13.70	45.10	14.90
Moysich (2000)	Breast	White	Cases	HDL	53.00	14.00	54.00	15.00	49.00	12.00
Moysich (2000)	Breast	White	Controls	HDL	57.00	18.00	54.00	18.00	50.00	13.00
Cibeira (2014)	Breast	Latinos	Cases	LDL	166.30	56.40	109.70	51.40	106.50	56.40
Cibeira (2014)	Breast	Latinos	Controls	LDL	110.60	35.40	100.70	31.30	135.70	50.90
Trompet (2009)	All types	White	Both	LDL	128.77	28.18	146.95	32.52	154.68	29.23
Souza (2009)	Colorectal	Latinos	Cases	LDL	122.60	0.57	118.90	48.50	109.10	32.40
Souza (2009)	Colorectal	Latinos	Controls	LDL	93.20	23.60	143.90	54.10	121.00	25.70
Moysich (2000)	Breast	White	Cases	LDL	124.00	43.00	143.00	41.00	159.00	58.00
Moysich (2000)	Breast	White	Controls	LDL	126.00	39.00	153.00	40.00	152.00	49.00
Cibeira (2014)	Breast	Latinos	Cases	TC	253.50	72.50	189.90	55.20	194.70	50.30
Cibeira (2014)	Breast	Latinos	Controls	TC	199.50	35.20	204.80	42.30	223.60	51.80
Trompet (2009)	All types	White	Both	TC	206.50	35.23	218.87	32.52	226.99	38.98
Souza (2009)	Colorectal	Latinos	Cases	TC	179.60	13.00	182.30	56.00	176.50	36.50
Souza (2009)	Colorectal	Latinos	Controls	TC	161.20	25.80	213.70	59.40	185.60	36.90
Moysich (2000)	Breast	White	Cases	TC	204.00	44.00	227.00	42.00	241.00	58.00
Moysich (2000)	Breast	White	Controls	TC	213.00	45.00	236.00	42.00	232.00	45.00
Cibeira (2014)	Breast	Latinos	Cases	TG	181.00	159.30	159.10	61.80	190.30	241.10
Cibeira (2014)	Breast	Latinos	Controls	TG	158.10	91.30	153.20	83.10	188.80	122.80
Souza (2009)	Colorectal	Latinos	Cases	TG	134.00	10.50	107.60	55.80	131.90	63.40
Souza (2009)	Colorectal	Latinos	Controls	TG	106.00	67.40	132.60	61.60	109.80	24.80
Moysich (2000)	Breast	White	Cases	TG	136.00	77.00	146.00	95.00	161.00	129.00
Moysich (2000)	Breast	White	Controls	TG	154.00	90.00	147.00	113.00	153.00	143.00

Abbreviations: HDL, high-density lipoprotein; LDL, low-density lipoprotein; TC, total cholesterol; TG, triglyceride; SD, standard deviation.

**Table 3:** Subgroup analysis of *APOE* gene ε2/ε3/ε4 polymorphism with cancer risk

Subgroup	No. of studies	ε2 versus ε3				ε4 versus ε3			
		OR	95% CI	P	*I*^2^	OR	95% CI	P	*I*^2^
Cancer type									
Breast cancer	10	0.95	0.78–1.17	0.654	0.0%	1.18	0.95–1.48	0.132	42.8%
Colorectal cancer	8	1.05	0.95–1.21	0.277	0.0%	0.96	0.82–1.14	0.667	50.4%
All cancers	3	0.96	0.81–1.13	0.617	32.9%	1.02	0.77–1.36	0.088	80.5%
Ethnicity									
Caucasian	14	0.94	0.81–1.08	0.389	22.8%	0.92	0.81–1.03	0.135	33.1%
Asian	4	1.01	0.69–1.47	0.969	34.7%	1.40	1.00–1.94	0.047	37.6%
Latinos	4	0.67	0.41–1.09	0.109	0.0%	1.05	0.69–1.60	0.819	44.8%
Mixed	4	1.07	0.93–1.22	0.339	9.7%	1.05	0.85–1.31	0.649	74.2%
Source of controls									
HB	16	0.95	0.78–1.16	0.629	32.2%	1.05	0.86–1.28	0.661	64.8%
Population-based	10	0.98	0.91–1.04	0.434	2.4%	0.97	0.93–1.02	0.297	3.0%
Study design									
Retrospective design	23	0.97	0.86–1.11	0.679	20.0%	1.03	0.90–1.16	0.704	53.2%
Prospective design	3	0.96	0.81–1.14	0.639	36.7%	0.92	0.80–1.06	0.239	36.6%
Matched status									
Yes	10	0.97	0.90–1.05	0.455	6.5%	1.02	0.94–1.11	0.669	23.5%
No	10	0.95	0.74–1.21	0.648	38.9%	0.92	0.72–1.20	0.549	69.0%
NA	6	1.06	0.82–1.37	0.667	17.6%	1.01	0.77–1.32	0.971	55.8%
Total sample size									
<500	13	0.82	0.65–1.04	0.097	0.0%	0.97	0.75–1.25	0.808	58.0%
≥500	13	1.01	0.90–1.13	0.897	38.7%	1.01	0.92–1.11	0.863	50.5%

Abbreviations: OR, odds ratio; 95% CI, 95% confidence interval; NA, not available.
